# Supplementary material for: Optimizing Access to the COVID-19 Vaccination for People Experiencing Homelessness
Source: Int J Environ Res Public Health. 2022 Nov 25;19(23):15686. doi: 10.3390/ijerph192315686 (PMC9736191; doi:10.3390/ijerph192315686)
Supplement: Supplementary file 1 [file ijerph-19-15686-s001.zip › ijerph-1985340-supplementary.pdf]

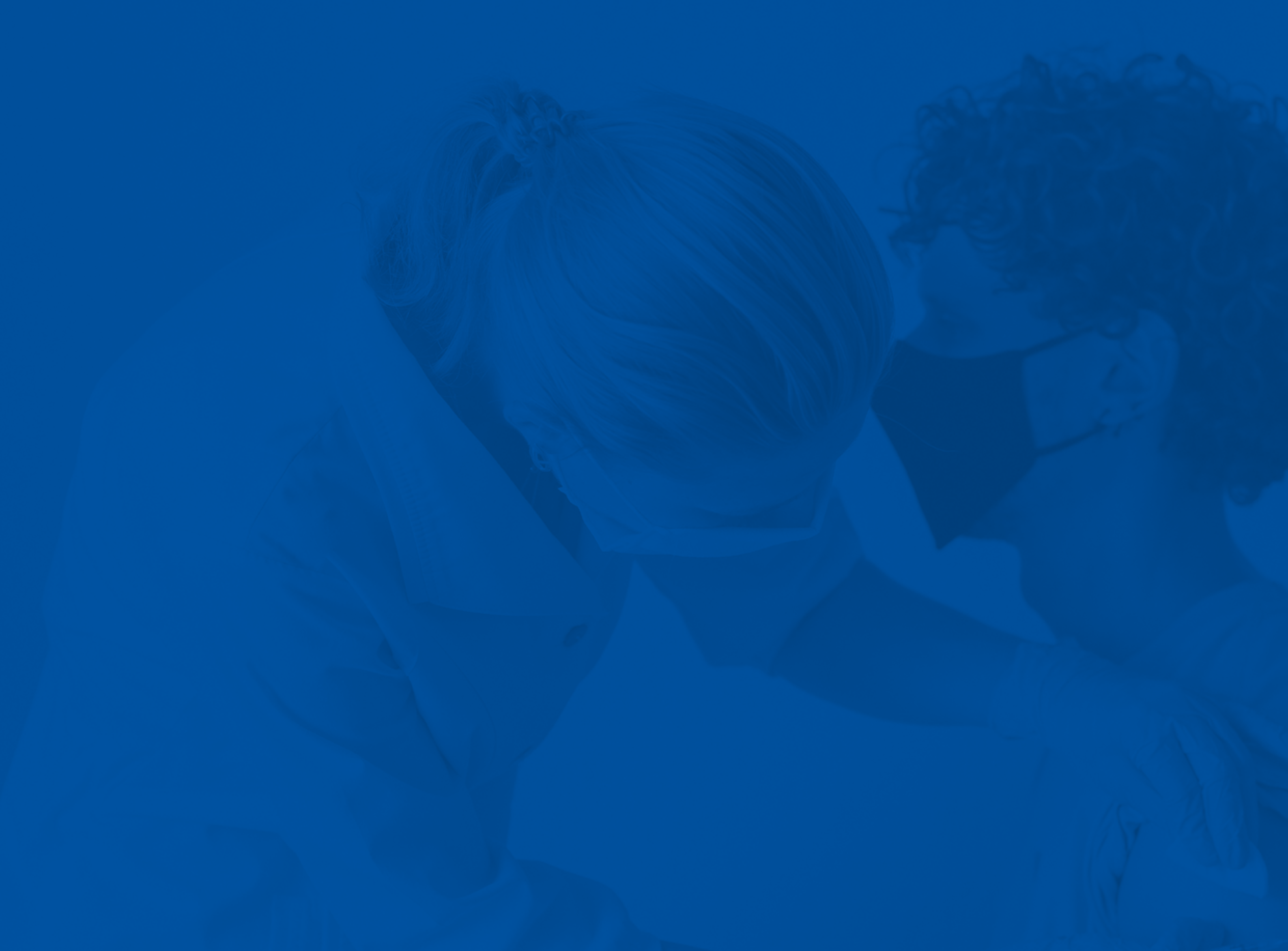

BLUEPRINT MODEL OF CARE TO SUPPORT VACCINE HUBS

FOR PEOPLE EXPERIENCING HOMELESSNESS

PURPOSE

The purpose of this document is to offer guidance to other agencies in the development and delivery of vaccine hubs for people experiencing or at risk of homelessness. The model of care described here is also relevant to vaccination efforts targeting other hard to reach or marginalised people and communities.

The contents of this blueprint are based on the experiences of those involved in the Inner City COVID-19 Vaccine Hub at the Ozanam Learning Centre, St Vincent de Paul Society NSW. A summary of the key aspects of the Vaccine Hub are provided below including the stakeholders involved, the model of care, and the equipment and training required.

Associate Professor Jane Currie, Queensland University of Technology, led a research collaboration with St Vincent's Hospital Network Sydney, to establish the evidence base to underpin the Vaccine Hub model of care presented in this document.

DEVELOPMENT OF THE VACCINE HUB

The Inner City COVID-19 Vaccine Hub was established in May 2021 to improve access to vaccination for people sleeping rough, people in specialist homelessness services and people at risk of homelessness, such as those living in social housing or temporary accommodation (NSW Health & St Vincent's Health Network Sydney, 2021). The Vaccine Hub was initiated by stakeholders at St Vincent's Hospital Sydney, Matthew Talbot Primary Health Clinic (located within the Matthew Talbot Hostel crisis accommodation service). South Eastern Sydney Local Health District Homeless Health Program Manager, and the Kirketon Road Centre (a walk-in primary health care service in Kings Cross). Engagement was sought from health and non-health partners within inner city Sydney to establish a collaborative approach. This included partners delivering health, social, and community services to people experiencing poverty and other forms of vulnerability. The St Vincent de Paul Society's Ozanam Learning Centre — a community centre located next to the Matthew Talbot Hostel, Woolloomooloo, which ordinarily provides opportunities for people in the target client group to connect, learn, participate in activities, and access support — was identified as a suitable location for the hub.

A process for the sharing of resources, such as Accredited Nurse Immunisers, consent forms and a common approach to messaging was established. Stakeholders committed to the Inner City COVID-19 Vaccine Hub included St Vincent's Health Network, South Eastern Sydney Local Heath District, Kirketon Road Centre, Matthew Talbot Primary Health Clinic, Sydney Local Health District, City of Sydney Council, Department of Communities and Justice, Neami National and St Vincent de Paul Society NSW. The first Vaccine Hub was implemented on Thursday 20th May 2021, at the Ozanam Learning Centre, and has run weekly since. As at mid-September 2021, more than 5000 people had been vaccinated against COVID-19 via the Vaccine Hub.

STAFF MEMBERS INVOLVED IN DELIVERING THE VACCINE HUB

The Inner City COVID-19 Vaccine Hub involves a broad spectrum of staff members.

Key medical personnel included Registered Nurses who are Accredited Nurse Immunisers (and who had completed the online training requirements) to administer the vaccines, and a General Practitioner to discuss the vaccines, assist people to complete the vaccination consent form and support any potential adverse reactions.

Peer Support Workers and Aboriginal Health Workers promoted a culturally appropriate environment.

Administrative support staff logged patient attendance and collated consent forms on the day, and sent texts and made follow-up calls during the week.

Members of other services were in attendance at the Inner City COVID-19 Vaccine Hub to provide support with housing and access to social services.

The managerial roles were operational in terms of ensuring adequate resourcing, engagement of other services, and then frontline in terms of managing the flow at point of vaccine administration. Input from Pharmacists was critical to ensuring appropriate cold chain management of the vaccines.

MODEL OF CARE

The Inner City COVID-19 Vaccine Hub hours of operation are every Thursday between 10am-2pm. The Vaccine Hub adopted the following practices:

- **Person-centred**
  A high degree of flexibility meant the experience could be tailored to an individuals' need, especially where people experienced barriers to access.
- **Trauma informed**
  The partners' prior experience working with people experiencing homelessness meant they were trained to recognise the signs and symptoms of trauma, could respond appropriately, and ensure the experience was not retraumatising.
- **Collaborative care**
  The model valued the provision of both health and social care, delivering the vaccine as part of a service that recognised the broader context of people's lives.
- **No wrong door**
  Anyone who presented at the Vaccine Hub was provided with information they needed to access a vaccine, even if a vaccine could not be provided via the Vaccine Hub, on the day. On some occasions this included supporting people to navigate the online booking system or connect to another service.

  "We know from working with vulnerable communities, they are so much more at risk than any other population. We see that day-to-day and we know how difficult it is for people experiencing things like trauma and homelessness. The book-in systems don't work, people don't have access to technology. It's important that there's a really flexible, open-door approach. How do we create something that's accessible for vulnerable communities and make sure that they are protected throughout the pandemic as well." (Stakeholder 12)

PHILOSOPHY OF CARE

The philosophy of care was one of teamwork, collaboration, and partnership around a common goal *'…there was no ego involved…'* (Stakeholder 2).

The goal was about improving access to vaccination by *"…taking healthcare to the people…"* (Stakeholder 1).

There was a *"...public health commitment... we need to make sure people are vaccinated..."* (Stakeholder 10) that ensure that access was *'…equitable…'* (Stakeholder 12).

FLOW OF VACCINATION

The flow of the Inner City COVID-19 Vaccine Hub was reported as critical to its success. Ensuring that those waiting to receive the vaccine were well informed of the process, were aware of how long they had to wait and had the opportunity to ask questions.

An important component of achieving flow was making sure staff had clearly defined roles, and that these roles were identified on their clothing, which was usually a high visibility vest.

Given that people were waiting for over an hour to receive their COVID-19 vaccination, a medical officer, nurses, and Aboriginal Health Workers were available to those waiting, to answer any questions and to offer support. This gave people time to consider whether they were comfortable to have the vaccination or perhaps whether they would have it on another occasion, *"…it wasn't a production line…they could get the information they needed…it wasn't assertive. We were just there and if people wanted it they could have it…'* (Stakeholder 4).

Signage around the Inner City COVID-19 Vaccine Hub was important to identify members of the public who were eligible to receive vaccination and avoid people who were not experiencing or at risk of homelessness being turned away.

The specific flow of the COVID-19 Vaccine Hub was as follows:

1. Clients arrive at the Inner City COVID-19 Vaccine Hub and are met outside by Ozanam Learning Centre employees, who speak with all clients to ensure adherence to COVID-safety guidelines, determine eligibility, and check-in using laminated QR codes or paper-based sign-in forms.
2. Eligibility screening provides an initial opportunity to identify additional support needs and make referrals.
3. As people join the queue, they are provided with information about the process including expected wait times, and given the opportunity to ask questions.
4. As people wait in the queue, they are provided with access to water and healthy snacks (where Covid restrictions allowed) and masks. The availability of employees from the Ozanam Learning Centre and other partner agencies gives people additional opportunities to ask questions and connect with extra services.
5. As people move toward the front of the queue, staff are present and available to support clients receiving their first dose to complete their NSW Health consent forms; particularly people with low literacy or from non-English speaking backgrounds.
6. When clients reach the front of the queue they enter the waiting room. A medical practitioner from the Matthew Talbot Clinic is available to answer questions and assist clients receiving the first dose of the vaccine to finalise their consent forms.
7. Once paperwork is complete and a client's name called, they are greeted by a nurse and invited into one of the vaccination areas.
8. After receiving the vaccination, they move to a recovery area for the required observation period (15 or 30 minutes), where they are supervised by a doctor in this context.
9. Clients then leave the premises via a separate exit.

DIAGRAM OF INNER CITY COVID-19 VACCINE HUB

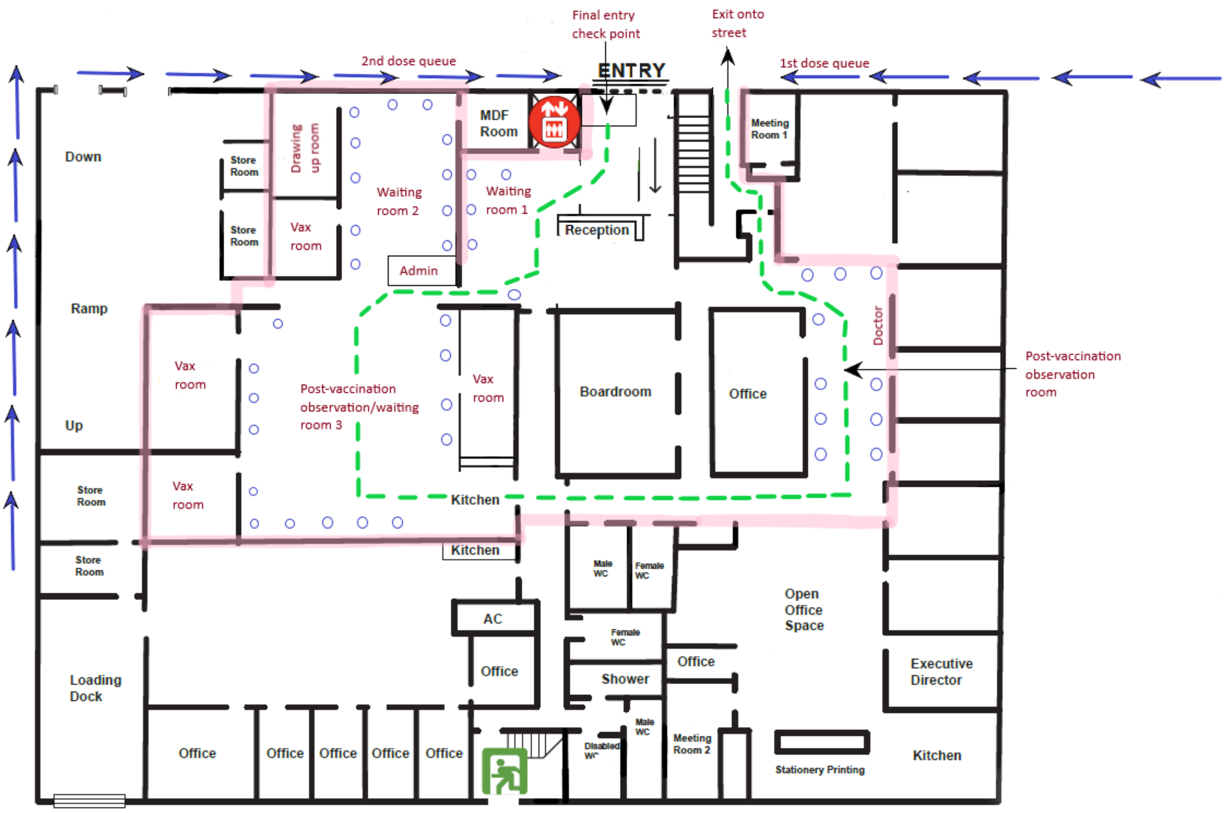

EQUIPMENT REQUIRED

The medical equipment required included the COVID-19 vaccinations, which were supplied by the St Vincent's Hospital Sydney Pharmacy in temperature-controlled esky bags with thermometers to ensure cold chain was maintained. Emergency resuscitation equipment is available from the Matthew Talbot Health Clinic adjacent to the Ozanam Learning Centre. An anaphylaxis kit was also available on site.

To facilitate the flow within the Vaccine Hub, NSW Health COVID-19 vaccination consent forms were readily available and laminated check-in QR codes were displayed throughout the entry area. COVID-safety marshal vests were worn by key members of staff responsible for patient flow, and of course face masks were made readily available to ensure compulsory mask wearing was adhered to. Other key equipment were bollards and signs to indicate the flow of movement through the Vaccine Hub.

A key advantage of holding the Hub in the Ozanam Learning Centre was that it was a building already used by members of the community. There was a sense that had a government building been used rather than a community-based building, people may not have engaged with the Inner City COVID-19 Vaccine Hub as readily. Nonetheless, the building was described as *'...still a white space...'* (Stakeholder 10), and although there were Aboriginal Health Workers and diversity among the healthcare and service providers delivering the Inner City COVID-19 Vaccine Hub, there was the possibility that not everyone was comfortable to go there. The location of the Hub was therefore extremely important.

SUCCESS FACTORS

Below the factors that appear to be facilitating the success of the Vaccine Hub are listed.

- **A common purpose**
  All partners contributing to the Vaccine Hub shared a common purpose; the wellbeing of people experiencing or at risk of homelessness. The majority had extensive experience working with the target client group and were focused on maximising access for people who would otherwise be likely to encounter barriers. No single agency had ownership of the Vaccine Hub.

  > *"We all had one single mind which was really to protect our community, to protect vulnerable people, to support people who we knew had found it more difficult to access healthcare."* (Stakeholder 4)

- **Shared resources**
  All partners contributed resources including time, skills, the venue, and equipment. Mutual agreement that vaccines were critical to protect people during the pandemic, and should be made available as quickly as possible, made it easier to mobilise or redirect internal resources towards a collaborative response.

- **Respect**
  The collaboration was premised on mutual respect for the skills, resources and experiences each partner brought to the table.

- **Communication**

  Clear and direct lines of communication were established and maintained between decision-makers in all partner organisations, making it possible for quick decisions and adjustments to be made in what became a fast-changing environment.

  > ***"Once we were like, "Okay, this is a system that works, we've figured out everything", there was still, in a sense, planning with it. At the beginning, planning meetings with all the partners the day before or two days beforehand every week. Plus, a briefing in the morning, and then a debrief in the afternoon."*** **(Stakeholder 10)**

- **Continuous improvement**

  Mini-debriefing sessions were held at the end of each day on which the Hub operated, with larger debriefs held weekly. A morning 'huddle' was held to brief all staff prior to each Vaccine Hub session. This gave all partners the opportunity to reflect on their experiences, identify issues, and refine the model accordingly.

- **Promotion**

  The Vaccine Hub's reach and impact was enhanced by using established networks and interagencies to promote the opportunity, and by leveraging existing branding and promotional tools. Promotion was ongoing and supported by strong buy-in from local services working with vulnerable people and/or communities[1], who then extended invitations to current and/or recent clients.

  > ***"So we had these flyers. We put them everywhere. We sent them everywhere. We distributed them through all the homelessness interagency groups that we had. And we took them on patrol. We had a lot of people who – in the first weeks in particular - were sleeping rough that got vaccinated."*** **(Stakeholder 4)**

- **Media**

  Expecting that the media would be interested in this initiative, they were proactively invited to attend. Positive coverage helped raise the Vaccine Hub's profile and promoted the opportunity to a broader audience.

- **Consistency**

  Clear and consistent messages about the availability of the vaccine at the Vaccine Hub — run at the same venue on regular days (weekly), with consistent hours and staffing — made it easier for people to understand the opportunity and how to access it. Over time, stories shared by people who had accessed the clinic helped build trust and confidence amongst others in the community who were initially reluctant to attend.

  > ***"We were just saying, "You can come in. This is really good. This is why you should have it. We'll be available. Don't worry if you don't want it. We'll have it next week." So it was assertive encouragement."*** **(Stakeholder 2)**

- **Relationships**

  Promoting the Vaccine Hub in partnership with local community service providers meant potential clients received an invitation to be vaccinated from a source with whom they already had a relationship of trust. Many of the organisations involved were signatories of the Intersectoral Homelessness Health Strategy/Alliance and *'…as much as the document is just a flashy document, what it has done is facilitate relationships and set expectations and obligations of us working together."* (Stakeholder 12). These services are often required to compete for funding, sometimes against each other, but in this instance they were connected around a common cause.

[1] Including people experiencing homelessness, people experiencing problematic drug or alcohol use, and people with disability.

This helped build confidence that the process would be manageable, and that any additional support needs could be addressed. On the day, the familiarity and comfort of staff with people accessing the service (and vice versa), and their capacity to respond flexibility to client needs, helped ensure clients felt safe and supported during the experience.

> *"We're working together for clients it doesn't matter which service is working with them…"* (Stakeholder 6)

- **Assertive outreach**
  Direct approaches were made to people sleeping rough or living in social housing communities. This extended the Vaccine Hub's reach to people not already linked to a service and presented an opportunity to allay concerns about the vaccine during one-on-one conversations, and *'…for a lot of rough sleepers, that was a better engagement space…'* (Stakeholder 11) than them attending the Ozanam Learning Centre.

- **Person-centred approach**
  The partnership between health and social services made it possible to identify and address barriers to accessing the vaccine. Staff at the Ozanam Learning Centre and/or case workers from local service providers were able to identify barriers (e.g. barriers experienced by people with mental health issues, social anxiety, cognitive impairment, backgrounds of trauma, physical disability, and low literacy levels) and work collaboratively with health professionals to address these barriers (see box 1 for examples).

  > *"We weren't on an appointment system…we had behaviourally challenged people who were hiding under tables…we were able to offer the time and just have that chat and it worked really well…"* (Stakeholder 9).

  There were spaces within the Vaccine Hub where people could sit and think and take their time before deciding on whether to receive the vaccine or not *"We had a space…a quiet space to sit in for a while…sometimes people just need a bit of quiet space with somebody that they trust…"* (Stakeholder 8).

- **An experience not just a service**
  Partners focused on ensuring the Vaccine Hub would deliver a positive experience for people accessing the service, maximising the likelihood people would return for their second dose. Considerations included music and atmosphere, comfort, and most critically, the mindset of all employees working in the Vaccine Hub *"….it's a place people feel safe and have existing relationships…people felt it was set up for them…"* (Stakeholder 5). When COVID restrictions allowed, personalised touches such as a welcome barbeque, colouring-in activities for children, or the provision of gift bags made by members of the community were included as part of the experience.

- **An opportunity for connection**
  Time spent at the Vaccine Hub — waiting for the vaccination or during the post-vaccine observation period — created opportunities for staff to check-in with clients, speak about their experiences during COVID and offer additional support and services. Some additional services were offered on site, although these varied over time and in response to COVID restrictions. (See box 2 for examples).

  > *"That element of coming together in a safe space, we're all in this together, was something that we quite enjoyed. Because a lot of people found – like we all did, but clients in particular found COVID so isolating, and so lonely."* (Stakeholder 6)

CHALLENGES AND CONSIDERATIONS

The establishment of the Vaccine Hub encountered a series of challenges, some of these were short-lived and were resolved through the dynamic development of the model of care, others were enduring, as follows:

- **Cultural safety**
  Ensuring translated versions of communication materials were readily available, and ensuring access to interpreters onsite, would have improved accessibility for people from non-English speaking backgrounds. The presence of Aboriginal Health Workers was critical *"…one of the most important things for Aboriginal people to talk about vaccination is the Aboriginal Health Workers…"* (Stakeholder 4). To increase their approachability all Vaccine Hub staff wore T-shirts with Aboriginal symbology.

  > *"We worked with our Aboriginal partner organisations, and I think having those relationships helped overcome some of the hesitancy…"* (Stakeholder 11)

- **Resources**
  The Vaccine Hub consumed considerable time and resources. Where employees were redeployed to work at the Vaccine Hub, for some partners this necessitated reprioritising existing workloads. Should these efforts be replicated in other jurisdictions or locations, they would be enhanced by access to additional resourcing and/or a commitment to flexibility in the delivery of contracted services from funding bodies.

  > *"We had to take nurses out of the other duties and it was very difficult… it was a juggle. At one point, we were having about five of us down there a week and that was challenging."* (Stakeholder 4)

- **Queue management**
  Ensuring people maintained social distancing while waiting in the queue was challenging, particularly in the initial weeks when there was high demand. Ensuring employees were able to engage with people in a friendly and positive manner, and providing them with COVID-safety vests as a visual cue, made this task easier. Employees were also able to identify people whose mobility, health, anxiety or behavioural issues made waiting challenging, and fast-track these individuals. Screening for homelessness and for those awaiting a COVID-19 test result was also important *'… obviously we don't want to vaccinate someone who's got COVID…"* (Stakeholder 4).

- **Community impact**
  Lines of people queuing along the street as they waited for a vaccine had a significant impact on residents and others in the community. Concerns could have been alleviated earlier with proactive communication prior to establishing the Vaccine Hub. Over time, the local community became more involved,

  > *"…some of the residents and the clients, they were helping out in terms of just getting things set up, stuff like getting food ready and all that sort of stuff. I think there was a sense of ownership on that space as well."* (Stakeholder 11)

- **Signage and information**
  Ample signage was needed to ensure information was available to people wherever they were in the queue. Where possible, signs were marked out the day prior to each Vaccine Hub to accommodate people arriving early. When necessary, to help ensure people did not spent unnecessary time waiting in the queue, a loudspeaker was used to make announcements about eligibility.

- **Clarity of messaging**
  It was important that information promoting the Vaccine Hub and on signage used at the venue was clear and unambiguous — particularly in relation to eligibility criteria — to minimise the potential for misinterpretation.

- **Eligibility screening**
  In the early weeks of the Vaccine Hub, when demand for the Pfizer vaccine exceeded supply, limiting access to the Vaccine Hub to people who met the eligibility criteria was challenging. Ensuring employees were equipped with information and strategies to politely decline people who did not fit the eligibility criteria was important.

- **Minimising wastage**
  To avoid wastage, all Pfizer doses had to be used by the end of each day. As it was not possible to accurately predict how many people would attend the clinic, where excess doses were available employees reached out to eligible people in nearby communities, inviting them to the clinic.

  > *"There was a lot more planning that needed to go into the COVID vaccine because they're not pre-packaged vaccines like a Fluvax. A multidose vial presents its own problems…it was such a precious thing…"* (Stakeholder 1)

EXAMPLES

In Boxes 1 & 2 below, we provide examples of how the model of care optimised access to the COVID-19 vaccination and to other services.

BOX 1

Flexibility from all parties meant the experience at the Vaccine Hub could be tailored to individual needs. Examples include:

- Making arrangements for people with significant anxiety or trauma backgrounds to bypass the queue, including setting aside specific times early in the day or during the quieter lunch period.
- Making a separate waiting area available to people for whom the main waiting room presented challenges. At different times this included people with mental health issues or trauma backgrounds, people with young children, people concerned about their safety (for example, women who had left violent relationships), and people with significant disability who were unable to sit or wait for any period of time.

Further, working in partnership with local service providers allowed for the provision of additional supports. For example:

- A service supporting people with significant disability shared easy-read social stories with clients prior to vaccination. These stories were then provided to nurses at the Vaccine Hub to ensure consistency and familiarity.
- Some services provided transport for clients where this was otherwise not available. Where needed, case workers accompanied clients to the Vaccine Hub, providing social support.
- Where people required a friend or pet for comfort, this was able to be accommodated.

BOX 2

As clients queued for the vaccine, this presented an opportunity to connect with clients and offer other services, referrals and supports. For example:

- Existing clients who had become isolated during Covid were able to reconnect with services, receive up-to-date information about programs running during Covid (for example, social connection programs, narcotics anonymous), and be assisted to access these programs (e.g. shown how to use zoom).
- People who had not previously visited the Ozanam Learning Centre were offered information about the programs on offer, as well as appropriate referrals. We expect that for some people this experience will make it easier for them to visit the service in the future.
- When speaking with people to determine eligibility, people who had recently become homeless or who were at risk of homelessness were asked what supports they had in place. When Department of Communities and Justice was in attendance, they were able to offer supports such as access to Temporary Accommodation or assistance completing housing applications.
- On several occasions, the Kirketon Road Centre set up a pop-up clinic offering onsite dried blood spot testing for Hepatitis C.

CONCLUSION AND POINT OF CONTACT

The model described here has facilitated access to the COVID-19 vaccine for people who are marginalised and often considered hard-to-reach. Elements of the model will be useful in ongoing efforts to increase vaccination rates in response to the COVID-19 pandemic, administer booster shots, and in the roll-out of other vaccination initiatives.

Further, the relationships forged between partners involved in delivering the hub will have benefits for client care beyond the life of the project.

If you have any questions relating to the model of care at the Vaccine Hub, please contact:

Matthew Larkin
Manager Homeless Health Service
St Vincent's Hospital Sydney
matthew.larkin@svha.org.au
